# Anorectal Tuberculosis Presenting With Multiple Abscesses and Fistulas in a Young Adult Male Patient With Alcohol Use Disorder: A Case Report

**DOI:** 10.7759/cureus.107821

**Published:** 2026-04-27

**Authors:** Megan Shen, Nadia Khan, Ana Moran, Ahmad Aldeiri, Erin Honsa

**Affiliations:** 1 School of Medicine, Creighton University, Phoenix, USA; 2 Department of Internal Medicine, Creighton University, Phoenix, USA; 3 Department of Medical Education, Creighton University, Phoenix, USA

**Keywords:** alcohol use disorder, anorectal tuberculosis, disseminated tuberculosis, extrapulmonary tuberculosis, miliary tuberculosis, mycobacterium tuberculosis, perianal fistula, rectal abscess

## Abstract

Gastrointestinal tuberculosis (TB) is an uncommon form of extrapulmonary TB, with anorectal involvement representing an exceedingly rare subset. We report a fatal case of disseminated anorectal TB in a 28-year-old male immigrant from Kenya with a history of alcohol use disorder, a recognized risk factor for TB reactivation and spread. The patient initially presented with rectal pain and perirectal purulent discharge. On evaluation, he was found to have a left perirectal abscess, a right ischioanal abscess, and multiple fistulas. Acid-fast bacillus staining of debrided anorectal abscess fluid revealed *Mycobacterium tuberculosis* complex, with additional evidence of pulmonary involvement on chest imaging and bronchoalveolar lavage, consistent with disseminated TB. The patient was initiated on anti-TB treatment with rifampin, isoniazid, pyrazinamide, and ethambutol. However, his condition rapidly deteriorated as he developed acute respiratory distress syndrome and septic shock, ultimately resulting in his death. This case highlights the rarity and potential severity of anorectal TB in disseminated disease. We also emphasize the importance of considering TB in patients with persistent or atypical anorectal infections, particularly those with epidemiologic risk factors such as prior residence in TB-endemic regions and alcohol use disorder.

## Introduction

*Mycobacterium tuberculosis* (Mtb) is an acid-fast bacillus most commonly associated with pulmonary disease and is a major public health concern due to it being the leading cause of death from an infectious disease worldwide. An estimated 1.7 billion individuals are currently infected worldwide, with an estimated 10.7 million new cases globally in 2024 [1].

While the incidence of tuberculosis (TB) had been decreasing since 2003, the COVID-19 pandemic led to an interruption in diagnosis, patient management, and treatment [2]. This has unfortunately led to record-high new cases. In the United States, approximately 13 million people are living with latent TB infection, often acquired from TB-endemic regions, with approximately 80% of the TB cases arising from reactivation of untreated latent infection [3]. In fact, by 2023, 76% of TB cases in the United States were attributed to foreign-born individuals from TB-endemic countries [4]. Although the risk of TB is highest within the first year of immigration, the risk continues to persist up to 20 years after arrival in the country. However, advances in diagnostic testing, effective latent TB treatment regimens, and strict infection-control practices have contributed to the reduction of new cases in the United States and other high-resource countries [5].

Mtb is acquired through repeated or prolonged inhalation of respiratory droplets and aerosols from an individual with active pulmonary TB. Multiple exposure events are required to establish disease, as most immunocompetent individuals clear the bacteria efficiently due to the activity of alveolar macrophages, or successfully contain the bacteria within the lungs in granulomatous tubercules [6,7]. Approximately 90% of immunocompetent individuals who do acquire the infection establish clinical latency and are not infectious. During this latency period, many individuals remain asymptomatic; however, the 10% who do develop active infection will experience symptoms such as fever and night sweats, weight loss, pleuritic chest pain, and a persistent cough with or without hemoptysis [8].

Chest radiography may appear normal during the initial stages of infection, but eventually cavitary lesions develop, which helps clinically diagnose TB infection. Immunologic testing is also available to help guide the clinician, which is vital for early detection, as culturing alone does not suffice due to the slow-growing nature of Mtb [9]. These assays include the tuberculin skin test (TST), where purified protein derivative tuberculin (non-Mtb) is injected under the skin, and 48-72 hours later, the size of the induration is measured. The other test, a TB Interferon-Gamma Release Assay (IGRA), is positive in an infected individual four to seven weeks after initial infection. The IGRA test has the added benefits over TST of not being affected by previous TB vaccination, being specific for Mtb, and not requiring a follow-up visit with a clinician for interpretation [10]. Also, acid-fast staining is commonly performed on sputum samples of patients suspected of having TB; however, multiple negative stains alone do not indicate latency or no infection. Therefore, diagnosis of TB requires a multidisciplinary approach, along with a thorough patient history [7].

Of individuals living with latent TB, 5-15% may experience reactivation in their lifetime, often presenting insidiously with nonspecific symptoms such as cough, fatigue, and weight loss [7]. The risk of TB infection and reactivation is influenced by both host and environmental factors, including immunological status, HIV infection, close contact with infected individuals, birth in a TB-endemic area, and living in overcrowded areas. Alcohol use disorder (AUD) has been shown to be an independent risk factor for both active and latent TB, relapse, and mortality, with an estimated 8% of global TB cases linked to AUD [11,12]. It is believed that AUD impairs alveolar macrophages and increases pulmonary oxidative stress, thus weakening lung immunity and promoting Mtb growth and escape from tubercles.

Miliary TB is a severe form of disseminated infection resulting from lymphohematogenous spread and typically presents with nonspecific constitutional symptoms such as fever, chills, anorexia, weight loss, and night sweats [13]. Anorectal disease caused by Mtb is an exceedingly uncommon subset manifestation of gastrointestinal TB, representing approximately 1% of gastrointestinal TB, which itself accounts for less than 1% of all TB cases [14]. Anal TB is one of the rarest documented gastrointestinal presentations. To our knowledge, as of 2025, there has been very few cases published on rectal TB [15-17]. The rarity of this specific form of disseminated TB, combined with the organism's lack of response to empiric antibiotics typically used for bacterial rectal infections, can result in diagnostic delays, increased multidrug resistance, and progression to severe disease.

Here, we present a rare and fatal case of anorectal tuberculosis in a young adult male, with no previous history of active or latent TB, who presented with rectal pain and discharge, ultimately found to be caused by Mtb. AUD is thought to have attributed to the extrapulmonary manifestation of his Mtb infection.

## Case presentation

A 28-year-old male patient who had immigrated from Kenya to the United States eight years prior presented to the emergency department with rectal pain and perirectal purulent drainage. He had no known history of any episodes of active TB, nor had he been diagnosed with latent TB. His past medical history was significant for AUD.

**Figure 1:**
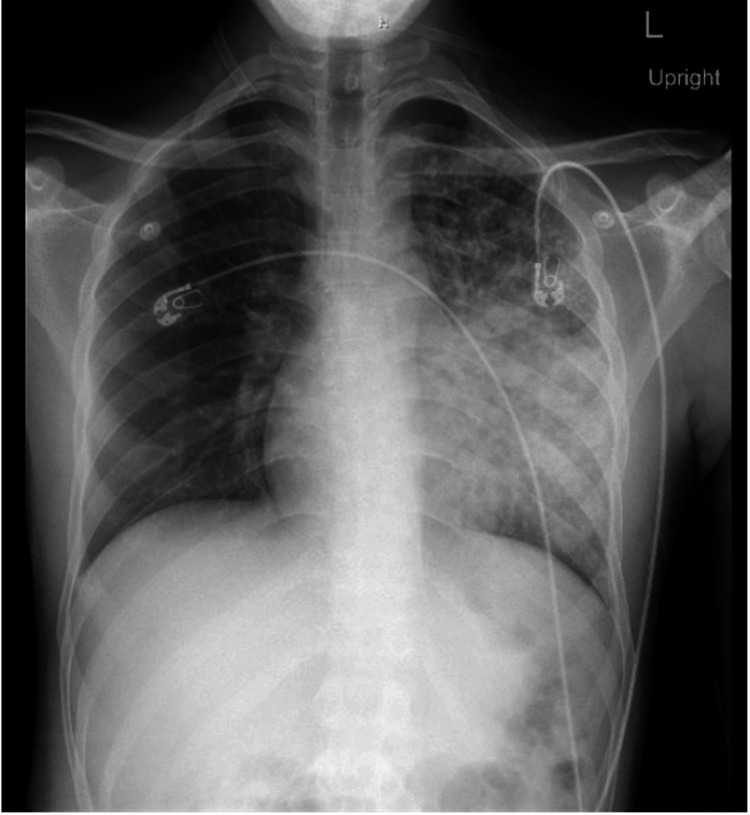
Chest X-ray at initial presentation, revealing diffuse airspace opacities throughout the left lung, as well as left-midlung consolidation.

**Figure 2:**
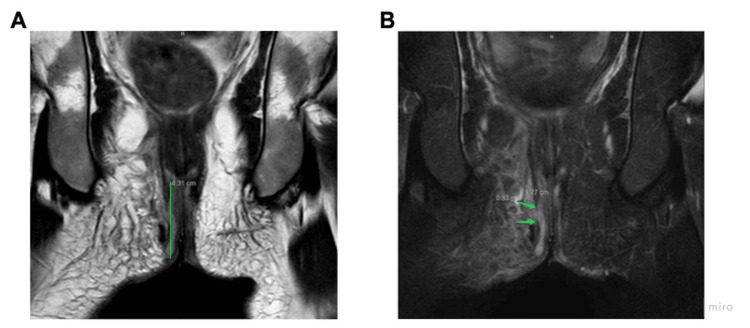
Pelvic MRI revealing (A) a perianal fistula (green line) measuring 4.31 cm, and (B) a rim enhancing collection measuring 1.3 cm x 0.33 cm (green arrows) with surrounding packing material.

At the time of admission, the patient met the criteria for sepsis and was hospitalized. Computed tomography (CT) imaging in the emergency department demonstrated a perirectal abscess. A chest X-ray revealed diffuse airspace opacities throughout the left lung. Left-midlung consolidation was concerning at this time for a possible infectious etiology (Figure 1). The patient was taken for operative evaluation, and a pelvic magnetic resonance imaging (MRI) scan performed prior to the procedure revealed a perianal fistula with fluid collection (Figure 2).

In the operating room, general surgery identified multiple abscesses, including a left perirectal abscess and right ischioanal abscess, along with several fistula tracts on examination under anesthesia. Both abscesses were debrided, and specimens were obtained for microbiological analysis. Empiric antimicrobial therapy with ceftriaxone, metronidazole, and fluconazole was initiated. Culture of the left perirectal abscess grew *Escherichia coli*, and an acid-fast bacilli stain of the abscess fluid was positive for Mtb complex (MTBC) (data not shown). Susceptibility profiles determined that the MTBC strain was sensitive to rifampin, isoniazid, pyrazinamide, and ethambutol. Based on the acid-fast results, a QuantiFERON-TB (IGRA) blood test (QIAGEN N.V., Venlo, Netherlands) was performed, which returned positive (data not shown). HIV testing performed at this time was negative. The patient was subsequently transitioned to appropriate targeted therapy for active TB, on rifampin (10 mg/kg), isoniazid (5 mg/kg), pyrazinamide (1500 mg/day), and ethambutol (1200 mg/day). It was decided not to administer corticosteroids to the patient during his admission.

Following the procedure, the patient became encephalopathic and remained febrile and tachypneic. Due to possible encephalitis, a lumbar puncture was performed. Opening pressure was normal, and cerebrospinal fluid culture was negative for TB during the remainder of his treatment and management. Repeated chest imaging demonstrated persistent left lung lobar consolidation, near complete lobar collapse, and cavitation within the large consolidation, which was concerning for pulmonary necrosis. Shortly after, the patient was transferred to the intensive care unit for acute hypoxic respiratory failure, eventually requiring intubation and mechanical ventilation. He developed acute respiratory distress syndrome and was placed on extracorporeal membrane oxygenation (ECMO). A bronchoalveolar lavage respiratory sample taken during this time was positive for MTBC using an acid-fast stain, and cultures grew *Enterobacter cloacae *complex. The patient continued to decompensate with worsening chest X-rays and continued ECMO dependence. A tracheostomy was performed, but the patient experienced significant bleeding at the tracheostomy site and remained hypoxic. The patient’s family was contacted and elected to transition the patient to comfort care. He passed away shortly after. 

## Discussion

Although Mtb infection most commonly presents as pulmonary disease, extrapulmonary TB can involve any organ system [13]. The gastrointestinal tract is the sixth most frequent site of extrapulmonary involvement, and it can result from swallowing infected sputum, hematogenous dissemination from pulmonary or miliary TB, or direct spread from adjacent organs [18]. While any portion of the gastrointestinal tract may be infected, anorectal involvement is very rare, and the limited number of published cases contributes to frequent delays in diagnosis [14-16]. Anorectal TB has been reported more often in males (ratio 4:1) and typically presents in their fourth decade of life [19]. This form of gastrointestinal TB is often associated with concurrent or previous pulmonary TB, which may not present with active pulmonary symptoms [20]. Perianal fistulas caused by Mtb represent the most frequent clinical manifestation of anorectal TB. However, it remains a small subset of extrapulmonary TB, and therefore, is often diagnosed late, with delay in appropriate treatment and patient management [21].

Because of the rarity and ambiguous presentation of anorectal TB, it is often mistaken for Crohn’s disease or malignancy, such as rectal adenocarcinoma [15,20]. Patients with anorectal TB may have prolonged rectal symptoms that do not improve with routine treatment or may present with systemic constitutional symptoms despite being otherwise young and immunocompetent. Standard treatment for anorectal TB by a drug-susceptible strain consists of rifampin, isoniazid, pyrazinamide, and ethambutol (RIPE) therapy for two months, followed by isoniazid and rifampin for an additional four months [22]. As noted with our patient, abscesses may also need to be debrided on a case-by-case basis. Most reported cases of isolated anorectal TB responded favorably to standard anti-TB drug regimens, with a low mortality rate; however, our patient presented late in the clinical manifestation of his extrapulmonary TB, marked by multiple fistulas, subsequent development of respiratory failure even after RIPE therapy had been initiated, and his unfortunate passing.

In our case, the patient was young with no other comorbidities; however, he did have a history of AUD and presented with a prolonged, untreated anorectal infection. He had immigrated from Kenya, a TB-endemic country [1], eight years earlier. Chest imaging and QuantiFERON-TB testing confirmed TB reactivation with pulmonary involvement and dissemination to the gastrointestinal tract. The patient’s initial symptoms, which included rectal pain and perirectal purulent discharge, were nonspecific and overlapped with common bacterial anorectal infections. A definitive diagnosis of tuberculous rectal abscess was only established after the debrided material underwent acid-fast staining. In addition, the patient was found to have a perianal fistula and multiple fistula tracts. A biopsy obtained from the patient’s fistula tract confirmed MTBC.

The patient’s presentation was unusually severe for his age and ultimately resulted in death, an outcome that is uncommon in isolated anorectal TB, even in the presence of complications. Most reported cases of anorectal TB involve localized disease that responds well to RIPE therapy, with low associated mortality [15]. As noted, AUD in a patient with latent TB increases the chance of both pulmonary reactivation and development of miliary disease, which aligns with our patient’s fulminant clinical course. AUD decreases the function of the immune system and has been specifically studied on how it reduces the body’s ability to effectively control and contain Mtb bacilli. Several innate immune responses that are required to control the growth of, or directly kill Mtb, are downregulated due to alcohol consumption. Tumor necrosis factor alpha (TNF-α), which is secreted by macrophages and ultimately increases phagocytic function, is dampened in the context of AUD [23]. This, coupled with decreased interferon gamma production and less nitric oxide (NO) response in the lungs, leads to early failure in the innate immune response to Mtb [24,25]. Even if the bacteria are successfully contained during granuloma formation, there is evidence that this response is dampened with AUD, and cell-mediated immunity in general is dysfunctional, including interleukin-2 production and CD4+ T-cell proliferation [25]. Therefore, defects in granuloma formation and T-cell maintenance and surveillance suggest that patients with AUD may have a higher likelihood of progressing to either reactivated pulmonary TB or extrapulmonary TB [23]. Our patient experienced both at only 28 years of age.

It is plausible that our young patients’ severe and fatal infection may have been exacerbated by AUD and the resulting immune system deficiencies. Our patient demonstrated poor response to RIPE therapy, and his condition was further complicated by concomitant bacterial infections, progression to sepsis, persistent pulmonary consolidation, development of acute respiratory distress syndrome, and eventual respiratory failure requiring ECMO. These factors likely contributed collectively to the fatal outcome of the disease.

## Conclusions

Anorectal TB is an exceedingly rare presentation of extrapulmonary infection with Mtb, leading to frequent misdiagnosis. The case we present illustrates the importance for physicians to consider the diagnosis of tuberculous anorectal infection in young patients with persistent anorectal or constitutional symptoms that do not respond to traditional antimicrobial therapy. This case also highlights the need to consider TB in patients with relevant risk factors, such as prior residence in endemic regions and a history of AUD. Although isolated anorectal TB typically responds well to RIPE therapy with low reported mortality, risk factors such as AUD carry a significantly higher risk of poor outcomes. Thus, early identification of TB as the underlying etiology of atypical anorectal abscesses or fistulas may allow prompt initiation of appropriate anti-TB treatment and reduce the likelihood of severe complications and fatal progression.
